# Multivariate mapping of ontogeny, taphonomy and phylogeny to reconstruct problematic fossil taxa

**DOI:** 10.1098/rspb.2023.0333

**Published:** 2023-05-31

**Authors:** Jane C. Reeves, Robert S. Sansom

**Affiliations:** Department of Earth and Environmental Sciences, University of Manchester, Manchester, UK

**Keywords:** ontogeny, taphonomy, early vertebrates, soft-tissue, fossil, non-metric multidimensional scaling

## Abstract

Exceptionally preserved fossils of soft-bodied organisms provide unique evidence of evolutionary history, but they are often contentious; different approaches frequently produce radically different reconstructions of taxa and their affinities. Conflict arises due to difficulties in disentangling the three non-independent factors that underlie all morphological variation within and between fossils: ontogeny, taphonomy and phylogeny. Comparative data from extant organisms can be extremely powerful in this context, but is often difficult to apply given the multi-dimensionality of anatomical variation. Here, we present a multivariate ordination method using discrete morphological character data from modern taxa at different ontogenetic and taphonomic stages (semaphoront and ‘semataphonts’). Analysing multiple axes of morphological variation simultaneously allows us to visualize character combinations that are likely to exist in fossil specimens at intersecting stages of growth and decay, and thus constrain interpretation of fossils. Application to early vertebrates finds variation in fossil specimens to be accounted for by all three axes: primarily decay in *Mayomyzon*, ontogeny in *Priscomyzon* and phylogeny in ‘euphaneropoids’ and *Palaeospondylus*. Our demonstration of empirical multi-factorial variation underscores the power of multivariate approaches to fossil interpretation, especially non-biomineralized taxa. As such, this conceptual approach provides a new method for resolving enigmatic taxa throughout the fossil record.

## 1. Introduction

The fossil record of exceptionally preserved soft tissues provides a unique perspective on the history of life on Earth. The complex interplay of biological and environmental conditions at various ‘konservat-lagerstätten’ [1] throughout time affords us valuable insights through the retention and preservation of tissues, such as skin, feathers and blubber, that can reveal otherwise unknowable morphologies and behaviours (e.g. [2,3]). Not only does this offer exquisite detail that would not be captured by the preservation of mineralized tissues alone, but it also provides evidence for the evolutionary history of entirely non-biomineralized organisms and communities (e.g. [4–7]). Even for those organisms that possess mineralized skeletal tissues, the early stages of their origin and diversification pre-dates readily fossilizable hard tissues [8]. However, the utility of the exceptionally preserved soft-tissue fossil record is limited by intrinsic difficulties and challenges.

Soft-tissue fossils can be incredibly hard to understand and often yield conflicting interpretations. While some are stunningly preserved, many are little more than shadows on the rock, hinting at the presence of anatomy but lacking clear definition or finer details that aid identification. The lack of recognizable features combined with the unusual trait combinations stem-taxa can display [9] results in fossils that are challenging to interpret in an evolutionary context [10]. Different approaches produce conflicting interpretations of both anatomy and phylogenetic affinity, leaving problematic taxa unresolved (e.g. [11]). Ultimately the crux of the problem is that fossil interpretation relies on one simple but elegant idea: knowing what anatomy is present and what is absent. However, this often proves difficult to achieve in practice.

Key to unlocking the evolutionary insights of fossil taxa is to make accurate and unambiguous interpretations of the suite of anatomical traits that are present and absent, as well as the homology and fidelity of those traits. Identifying morphological traits in fossils requires the ability to recognize both a feature in the fossil and its homology within other taxa. These are inextricably linked; homologies are needed to identify traits, but fossil traits are used to select the taxa to identify homologies with. Naturally, different comparator models lead to discrepancies in the interpretation of the same fossil structure, and concomitant variation in the resulting phylogenetic placement, meaning that care must be taken with their selection [10,12]. Absent anatomical traits are equally vital for positioning within a phylogenetic tree [10,11,13]. Recognizing absences should be a relatively straightforward task, but does not always prove to be. It is important to make the distinction between phylogenetic absences of traits (i.e. it was never there) and taphonomic absences (i.e. it was lost at some point after death) [10,13]. Furthermore, traits may be absent or modified because the organism is at an early developmental stage. To make these distinctions and recognize which traits are present (or absent), it is necessary to consider all the factors responsible for anatomical variation in all fossil taxa: ontogeny, taphonomy and phylogeny; ontogeny given that traits will be acquired, transform and occasionally be lost as an organism grows and matures, taphonomy given that traits will be lost or transformed to decay and decomposition before and during a panoply of preservation mechanisms, and phylogeny given that organisms from across the tree of life vary given their evolutionary history. Whereas ontogenetic and taphonomic changes occur during the life and death of an organism, creating variations between fossils of the same species, ‘phylogeny’ is distinctly different. ‘Phylogeny’, as used here, is the highly complex mix of evolutionary and biodiversity pressures, occurring over generations, that results in anatomical variations between species, rather than the individual members. Disentangling the factors and their influence is particularly difficult when interpreting the exceptionally preserved fossil record of soft-bodied organisms.

Difficulties in identifying and understanding the extent of the influence of each factor is often at the root of many of the debated and controversial taxa scattered throughout the fossil record. In the Devonian acanthodian *Triazeugacanthus affinis* [14,15], the ‘scaumenellization’ decay series has been reinterpreted as an ontogenetic sequence of transformation [16]. Whereas with the notorious *Palaeospondylus gunni,* known from thousands of Devonian fossil specimens, it remains unclear whether the small number of traits preserved in the fossils is due to taphonomically induced loss, or ontogenetic change, resulting in a huge number of proposed conflicting affinities and ontogenetic interpretations [17–20]. Difficulties like these are not constrained to just soft-tissue fossils. Even in one of the most iconic, well-studied fossils known, *Tyrannosaurus rex* [21], debates over ontogeny versus species or phylogenetic variation can be highly controversial [22–25]. While the different choices in selection of a comparator ‘bauplan’, i.e. understanding the influence of the phylogenetic factor of variance, result in the disagreements surrounding the affinities of infamous Carboniferous *Tullimonstrum gregarium* [26–28], and the enigmatic Cambrian vetulicolians, both of which appear to be firmly resistant to a clear, unambiguous phylogenetic placement [29]. Studies investigating the relationship between decay and preservation of anatomy have been useful in constraining fossil interpretation [30–33] but often, by necessity, focused on individual lagerstätte or taxa for which multi-faceted data are available.

Here, we present a new exploratory approach to objectively and quantitatively untangle the interdependent influences on fossil morphology. By using extant data, we blend traditional comparative palaeontological techniques with multivariate analyses to provide a comparative framework in order to aid interpretation of problematic fossils. This approach can be considered as equivalent to classical comparative methods, similar to comparing the morphology of the fossil specimens with an array of extant representatives at different ontogenetic and decay stages allowing the identification of similarities and diagnostic trait combinations. By using a phenetic approach, assessing the morphology variation between fossils in a framework defined by taxa of known phylogenetic affinity, it aims to visualize morphological similarities and dissimilarities. This in turn allows for the identification of trends in the drivers of variation, as well as an understanding of the impact of multiple factors, highlighted by the closeness of fossil specimens to the framework extant taxa, and highlight the potential biases in the preserved morphology that need to be included in interpretations.

Using early vertebrates as the case study group, we aim to show how our method is able to distinguish taxa on the bases of ontogenetic, phylogenetic and taphonomic factors. It therefore serves as a test of the approach of taking multiple sources of morphological variation into account simultaneously. By demonstrating this exploratory approach with a selection of previously studied taxa, we are able to highlight the impact of different comparison groups on interpretations, as well as need for caution when approaching morphological coding. Further expansions and applications of this method, including its use in other taxonomic groups are also discussed.

## 2. Methods

### (a) Case study group

Early vertebrates are a suitable case study given that extant vertebrates have well-characterized ontogenetic sequences of morphological change during direct and indirect development (e.g. [34–39]), a wealth of experimental decay data from representative taxa and ontogenetic stages (e.g. [9,40–42]), and the prevalence of problematic soft-tissue fossil taxa that have evaded classification (e.g. the ‘euphaneropoids’ [43–45] *Palaeospondylus* (e.g. [19,20]))

### (b) Combining axes of morphological variation

Each of the axes of morphological fossil variation (taphonomy, ontogeny and phylogeny) play an important role on their own, but they are also non-independent and can co-vary, interact and be conflated. The morphological impact of different influences can be easily mistaken for each other; as demonstrated by analysis of *Triazeugacanthus affinis* [16]. Patterns of anatomical growth and patterns of decay co-vary with phylogeny i.e. different taxa will have different axes of taphonomic and ontogenetic variation of morphology. As such it becomes increasingly hard to distinguish the cause of any anatomical variation seen; simply put, an absent character may be missing because it had not yet developed, it failed to be preserved, or it may have not evolved in the organism.

**Figure 1.**
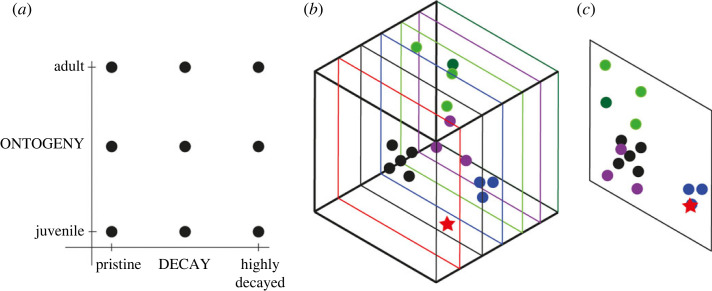
Theoretical model for the comparison of morphological variability in fossil taxa. (*a*) Ontogeny and taphonomy are two fundamental axes of variation that contribute to the fossil's morphology. (*b*) Combining data from known series of ontogeny and taphonomic effects from different modern and fossil species incorporates the influence of phylogeny on morphology. This creates a multidimensional framework analogous to morphospace or ecospace comparison approaches [47,49]. (*c*) Visualizing via reducing dimensionality (e.g. using multivariate ordination) allows comparison against the known intersection of each morphology influence and creates the potential to identify problematic taxa and the stage of development, or degree of taphonomic bias within their fossil, in a common framework.

Multivariate analyses are a well-established approach within biology and palaeontology [46], allowing visualization of multidimensional variability to identify patterns in data with applications ranging from comparing morphology [47,48] and ecological disparity [49,50], to its use for characterizing plants [51]. Here, we use a ‘morphospace’ approach [48], where the selected traits define the axes of a multidimensional space, containing all observed, and some potential, trait combinations (figure 1*a,b*) [52,53]. The relative positions in a morphospace can inform on the morphological disparity between taxa [47,54,55], but ordination techniques such as non-metric multidimensional scaling (NMDS), are required to summarize and visualize the complex variation within the multivariate datasets (figure 1*c*).

### (c) Establishing the axes of variation

#### (i) Ontogeny

Ontogenetic development can lead to different trait combinations dependent on age, varying from subtle differences to adult and juvenile stages that are not immediately recognizable as the same organism (e.g. the tadpole–frog metamorphosis [56]). Incorporation of the ontogenetic axes of morphological variation is relatively simple as the suite of anatomical traits accumulating and transforming through development can be observed and scored, from birth to adulthood, as a linear axis through time. This holds true even for complex developmental events such as metamorphosis, which occurs in an observable linear temporal path, even if the associated changes are non-trivial. To achieve this incorporation of morphology, we used a variation of the ‘semaphoront’ concept (Wolfe & Hegna [57], following Hennig [58]). Semaphoronts are individual ‘units’ within a species that essentially represent a ‘snapshot’ of an organism's morphology at different stages.

#### (ii) Taphonomy

Post-death, a myriad of taphonomic processes alter and distort anatomy that is eventually preserved. Fossilization occurs due to an exceptionally complex intersection of biological, geochemical and geological factors. Decay and decomposition are fundamental components of the taphonomic processes that lead to soft-tissue fossilization*.* While vital for preserving tissues (i.e. traits), they also lead to loss of characters and alteration of the carcass: controlling which tissues are able to be fossilized and reducing fossil fidelity [9,32,59]. Patterns of decay are therefore an important factor when interpreting the anatomy of fossils [11,31,32]. However, the precise impact of decay depends on the preservational pathways in operation at a particular fossil location [31], and subsequent geological processes can further alter and distort fossils [32,41,60]. Preservational pathways can interact [61] and differ between tissue type at the same site [62]. While tissue preservation can even vary between similar sites and locations [7], so the suite of anatomical traits present and absent in a fossil become hard to predict, and potentially unexpected biases can occur.

Clearly, incorporation of the taphonomic axes of variation is relatively difficult given the complex interactions between decay and preservation pathways. Here, we chose to focus on just one axis of taphonomic variation: loss of anatomical traits during decay. For entirely soft-tissue taxa, this is arguably one of the dominant taphonomic factors. Sequences of anatomical loss experimentally observed in extant taxa are the simplest case study, assuming a general pattern of enhanced preservation of relatively decay-resistant characteristics [31]. However, the techniques proposed here could also be modified to be applied to other models of preservation (e.g. instances where rapid decay might enhance preservation, for example, phosphatization of muscle tissue), and other taphonomic influences on morphology (e.g. transportation [63], sedimentology or environmental factors [64,65] etc.).

To capture the variation linked to decay, we adapted the semaphoront approach to codify ‘semataphonts’. In the sense that a semaphoront is defined as ‘the individual during a certain, however brief, period of time’ [57,58] (i.e. an organism at a particular ontogenetic stage), we define semataphont as a snapshot of an organism of following post-mortem processes. Here, we focus on semataphonts as incomplete organisms that have lost some anatomy to decay processes, but semataphont could also be applied to individual fossil specimens of the same taxon preserved under different pathways (e.g. pyritization versus phosphatization) or other taphonomic processes. For our analyses, semaphoronts were ‘divided’ into key decay stages (identified by Sansom *et al.* [12,41,42]), then the morphology for each resulting semataphont was assessed and scored to allow its incorporation in the analysis.

#### (iii) Phylogeny

The most fundamental underlying influence on morphological variation between taxa is phylogeny, i.e. different species have different combinations of morphological features given their evolutionary history. Fossil species are defined by the presence (and absence) of morphological characteristics. However, simple interpretations of the presence and absence of anatomical traits can quickly become non-trivial in the context of soft-bodied fossil taxa as a consequence of ontogenetic and taphonomic complexities, and the approaches used. Notwithstanding homology models, phylogenetic axes of morphological variation are relatively tractable given that anatomical suites of traits in different organisms can be observed and scored. To capture phylogenetic variation for early vertebrates, we used semaphoronts for a range of representative modern species: cyclostomes (lamprey and hagfish), gnathostomes (sharks) and non-vertebrate chordates (*Branchiostoma*). To investigate the problem of potential circularity in selection of an extant comparator [10], we followed the approach of Miyashita *et al.* [66] and Aldridge *et al.* [29] by creating several ‘versions’ for *Palaeospondylus* based on existing varying anatomical interpretations. The ‘versions’ represent five of the key hypotheses of affinity (cyclostome, gnathostome, hagfish, lungfish and shark) and the morphology was scored accordingly (further details are available in the electronic supplementary material [67]).

## 3. Data and data analysis

Extant taxa were divided into morphologically distinct ontogenetic stages (e.g. adult, juvenile etc.) to produce semaphoronts for the taxon. Semataphonts for each taxon were included to capture major changes associated with different stages of the post-mortem decay process at different ontogenetic stages. The final semaphoronts and semataphonts were treated as individual taxonomic units within the analysis, each representing the varying morphological traits present at different stages of ontogeny and decay. The presence (1) and absence (0) of key diagnostic and homologous traits for semaphoronts and semataphonts were compiled from literature (further details available in the electronic supplementary material [67]).

Our matrix of 71 character traits for 111 semaphoronts and semataphonts (table 1) was analysed in two different approaches with respect to treatment of fossil anatomy: coding style 1 allowed unknown character states (0/1/?) and forms the main basis for discussion here, while an alternative coding style 2 had a strict absence or presence only approach (0/1). An additional matrix was created that only included data from the extant taxa. These matrices were first converted to a NEXUS format, then a pairwise dissimilarity matrix was calculated using Gower's coefficient [68] with the package Claddis (v. 0.6.3) [69]. To allow visualization of the multivariate dataset, separate non-metric multidimensional scaling (NMDS) was performed on each distance matrix. An additional NMDS analysis was performed on a subset of the data that only included extant data. Principal coordinates analysis (PCoA) was also performed on the Gower distance matrices with Cailliez correction applied, as recommended to remove any negative eigenvalues that may occur due to missing data or non-Euclidean distances [70] using the R package ape (v. 5.4.1) [71]. The resulting scores from the NMDS and vectors from the PCoA were used to produce bivariate morphospace plots with the package ggplot2 (v. 3.3.2) [72]. All analyses were performed with R (v. 4.0.3) using the R studio software (v. 1.1.463) [73].

**Table 1.** List of number of semaphoronts and semataphonts used in analysis. Full details and references are available in the electronic supplementary material.

	ontogenetic stages (semaphoronts)	decay stages (semataphonts)	total
hagfish	adult	decay states 1–6 (adult)	7
lamprey	ammocoete, metamorphic stages 1–7, adult	decay stages 1–6 (adult)	21
		decay stages 1–6 (ammocoete)	
shark	embryo, pre-hatchling	decay stages 1–6 (embryo)	14
		decay stages 1–6 (pre-hatchling)	
*Branchiostoma*	adult	decay stages 1–6 (adult)	7
*Myxinikela*	fossil specimen		1
*Tethymyxine*	fossil specimen		1
*Mayomyzon*	fossil specimens of varying completeness		22
*Priscomyzon*	fossil specimens of varying completeness		7
*Mesomyzon*	two fossil specimens (adult, subadult)		2
‘euphaneropoids’	six extinct genera: *Lasanius, Jamoytius, Ciderius, Achanarella, Cornovichthys* and *Euphanerops* (multiple specimens of varying completeness)		24
*Palaeospondylus*	one extinct genus interpreted with different models of homology [17,18,20,66]		5
	total		111

## 4. Results

**Figure 2.**
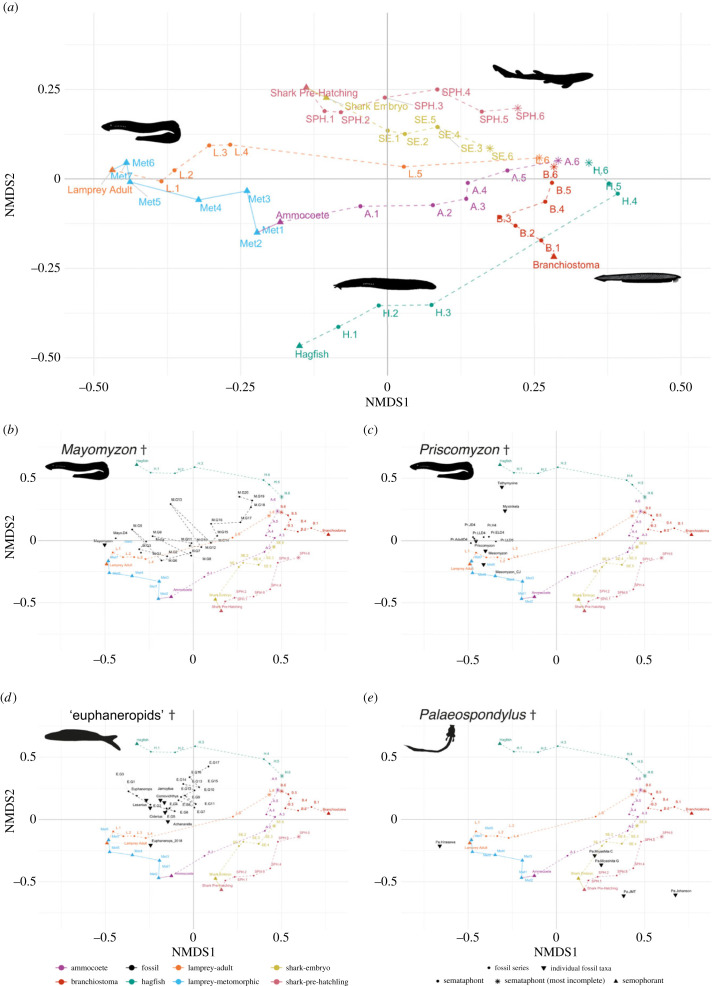
Morphological variation visualized in non-metric multidimensional scaling (NMDS) plots. (*a*) Dataset consisting of only extant taxonomic groups, (*b–e*), plots of an NMDS analysis including all taxa, with selected fossil groups plotted (coding style 1 incorporating uncertain entries as missing). (*b*) *Mayomyzon*, (*c*) *Priscomyzon* and *Mesomyzon*, (*d*) ‘euphaneropoids’, (*e*) *Palaeospondylus*. Dotted lines illustrate the temporal direction of inferred decay stages. Solid lines show direction of ontogenetic change. Silhouettes of *Palaeospondylus* and ‘euphaneropoid’ created for this image, remain images from Phylopic.org distributed under public domain licence. † indicates extinct groups. Higher resolution versions of all individual plots (electronic supplementary material, figures S1–S10) and information on specimens and individual labels can be found in the electronic supplementary material [67].

The NMDS ordinations reflect the variation seen within the dataset. The stress values for both coding systems in two dimensions were moderately high (= 0.220 and = 0.165, respectively), but they are close to, or under, the commonly accepted threshold value (0.2) [74,75]. Additionally, the Shepard stress plots (electronic supplementary material, figures S11 and S12) demonstrate the goodness of fit of the NMDS solutions, suggesting they represent the dissimilarity matrix distances well [75]. Conversely, each PCoA axis only accounted for a small amount of the total variation seen, so a large number of dimensions would have been required to adequately assess the data. Nevertheless, the general patterns of occupancy seen within bivariate plots of the first two PCoA axes (electronic supplementary material, figures S14, S16 and S18) were similar to the patterns seen within the NMDS plots (figure 2; electronic supplementary material, S1–S3). Due to this similarity, and the perceived ‘better fit’ of the NMDS to the data, only the NMDS morphospace plots have been considered further within this manuscript, the PCoA plots and associated data are available in the electronic supplementary material [35]

### (a) Extant taxa

The undecayed, adult morphologies of each modern taxon are at the extremities of the morphospace (figure 2*a*). Within each extant taxon, individual semataphonts and semaphoronts show very little overlap, with only a small number being indistinguishable (e.g. some lamprey metamorphic stages). The relative positions of semaphoronts and semataphonts of modern species are generally stable, but when fossil taxa are added to the framework (figure 2*b–e*), NMDS axis 2 is inverted for hagfish and the sharks. Ontogenetic sequences are clearly captured in terms of their morphological variation. For example, the successive development stages of lamprey show a continuum from ammocoete to adult in the morphospace. This is driven by morphological changes associated with a shift to a predatory lifestyle, including development of keratinized teeth and the loss of filter feeding traits such as buccal cirrhi, alongside the development of sexual organs (these changes are universal in lamprey, even in the non-predatory species [35]). The increasing incompleteness due to decay (indicated by the semataphonts stages 1–6) causes semataphonts from different taxa to become positioned closer together as they lose characters, starting with the most liable such as brains and other internal organs, and some cartilaginous traits [12,42] (figure 2). The later, more incomplete decay stages of each taxon group converge in an area in the top right quarter of the plot, united by the retention of several decay-resistant traits including myomeres, pigmentation and mineralized tissues [12,42]. To emphasize this pattern, the semataphonts have been connected in the plots within taxon groups (figure 2, dotted lines). In a general sense, the most complete, pre-decay semaphoronts occupy the extremities of the morphospace, and as anatomical traits are lost to decay, semataphonts occupy positions closer to a common point in the morphospace.

### (b) Fossil taxa

Comparison of the NMDS plots with different approaches to the treatment of missing data (figure 2; electronic supplementary material, S1 and S2) reveals considerable shifts in the patterns of occupation of all fossil taxa. When unknown ambiguous character states are permitted (coding style 1), fossil taxa produce a pattern similar to the extant taxa, generally exhibiting a series with the most incomplete specimens towards the extremities, as seen in extant semataphonts. With a less tolerant approach, where only present or absence of a trait is permitted (coding style 2), the relative position between modern taxa are maintained, but the vast majority of the fossils group close to the area occupied by the highly decayed extant semataphonts, with only a few exceptions. The following discussion focuses on the distribution of fossil taxa in the first analysis where ambiguous character states are permitted (i.e. ? coded).

#### (i) Hagfish

The fossil hagfish *Tethymyxine* occupies a position in the NMDS plot (figure 2*c*) close to the extant adult hagfish semataphont that represents the morphology at the first stage of decay. *Myxinikela* occupies a similar position on the NMDS1 axis to *Tethymyxine*, but in a more ambiguous space between the extinct lamprey and hagfish, near to the grouping of ‘euphaneropoid’ specimens.

#### (ii) Lampreys

The fossil lamprey specimens are recovered as morphologically close to the adult extant lamprey early decay stage semataphonts. Both *Mayomyzon* and *Priscomyzon* are known from multiple fossil specimens that have been variously interpreted as different ontogenetic stages and taphonomic stages relating to differing levels of anatomical completeness. Here, the *Mayomyzon* and *Priscomyzon* semataphonts (i.e. individual fossil specimens) are positioned closest to the extant adult semaphoront lamprey, while the *Mesomyzon* semataphonts are recovered close to the early decay stage extant adult semataphonts.

The *Priscomyzon* fossil semataphont specimens previously interpreted as a range of ontogenetic stages [76] are all positioned near to the semataphonts representing decay stages of extant lampreys. The fossil specimens interpreted as later *Priscomyzon* ontogenetic stages (juvenile and adults) are positioned closer to the extant adult lamprey semaphoront, and an early decay stage semataphont (stage 1) (figure 2*c*). This pattern is only disrupted by the late-larval *Priscomyzon* semataphont considered to be heavily decayed (decay stage 5), which is found nearer to the earlier ontogenetic stages of the other *Priscomyzon* specimens (while the less decayed late larval semataphont is closer to the adult specimen.

Fossil *Mayomyzon* specimens comprise a morphological series that runs along the axis of decay-related variation observed in modern lamprey, with a few clear deviations (figure 2*b*). A small number of *Mayomyzon* semataphonts, notably the specimen described as a late-larval stage with an advanced onset of decay (decay stage 4 (M.D4 [76]) (figure 2*b*)) occupy the same area as the early ontogenetic *Priscomyzon* specimens. There is a separation between the specimens described by Miyashita *et al.* [76] (largely interpreted as ontogenetic variation) and those from Gabbott *et al*. [31] (interpreted largely as taphonomic variation, driven by decay and preservational factors). While the Miyashita ‘ontogenetic’ specimens sit close to the extant adult semaphoront and *Priscomyzon,* the Gabbott specimens create a similar ‘path' to the extant adult semataphonts, located between the third and sixth extant decay stages. The Gabbott series can be roughly broken into three groupings. Specimens 1–6 are located around the third and fourth extant semataphonts with some overlap into the early ontogenetic stage *Priscomyzon* semataphonts, and specimens 8, 7, 10 and 11 form a tight association between the fourth and fifth extant lamprey semataphonts with 14–16 close by. The final grouping consists of specimens 17–20, which occupy an area between the most decayed extant hagfish and lamprey semataphonts.

#### (iii) Euphaneropoids

*Lasanius*, *Jamoytius*, *Euphanerops*, *Ciderius*, *Achanarella* and *Cornovichthys* roughly group together in a position in the morphospace between the lamprey and hagfish (figure 2*d*). Although, the *Euphanerops* semataphont created from the morphological description of Chevrinais *et al.* [77] occupies a different area of the plot, close to the extant lamprey decay stage 4 semataphont. Aside from this specimen, there are two apparent subgroups within the ‘euphaneropoid’ jawless vertebrates: a loose grouping of *Euphanerops* and *Lasanius*, and a second associated grouping of *Jamoytius, Achanarella*, *Ciderius* and *Cornovichthys*.

As seen in *Mayomyzon*, the Gabbott series of *Euphanerops* specimens (interpreted by Gabbott *et al.* [31] as representing taphonomic variation, both as decay and preservation) create a ‘path’ from an area of low decay to high, although the final specimens occupy a space closer to the decay stage 3 extant hagfish semataphont, rather than the later stages.

#### (iv) Palaeospondylus

*Palaeospondylus* is exceptionally sensitive to change in the model used for identifying homology. The majority of the *Palaeospondylus* ‘versions’ are located in the bottom right of the plot (figure 2*e*). Interpretations of anatomy through the lens of homology with either cyclostome and gnathostome models (labelled as Pa. Miyashita C and G, respectively [66]) results in similar placement of *Palaeospondylus* within the region occupied by non-decayed shark semaphoronts. The Moy-Thomas (lungfish, labelled as Pa. JMT [17]) ‘version’ occupies an otherwise unique area in the plot, near to the gnathostomes (extant sharks) in the bottom right corner. The Hirasawa *Palaeospondylus* (interpreted as a hagfish, Pa. Hirasawa [18]) occupies another unique area away from other *Palaeospondylus* ‘version’, relatively close to the final extant metamorphic and adult lamprey stages. This position is most likely an artefact in the dis/similarity matrix resulting in a lack of the hagfish synapomorphies used to assign affinity within the fossil occurring in the character list of this study. Finally, the Johanson ‘version’ (chondrichthyan, Pa. Johanson [20]) unexpectedly occupies an unoccupied area furthest away from all other taxa.

## 5. Discussion

### (a) Visualizing decay, ontogeny and ‘stem-ward slippage’

**Figure 3.**
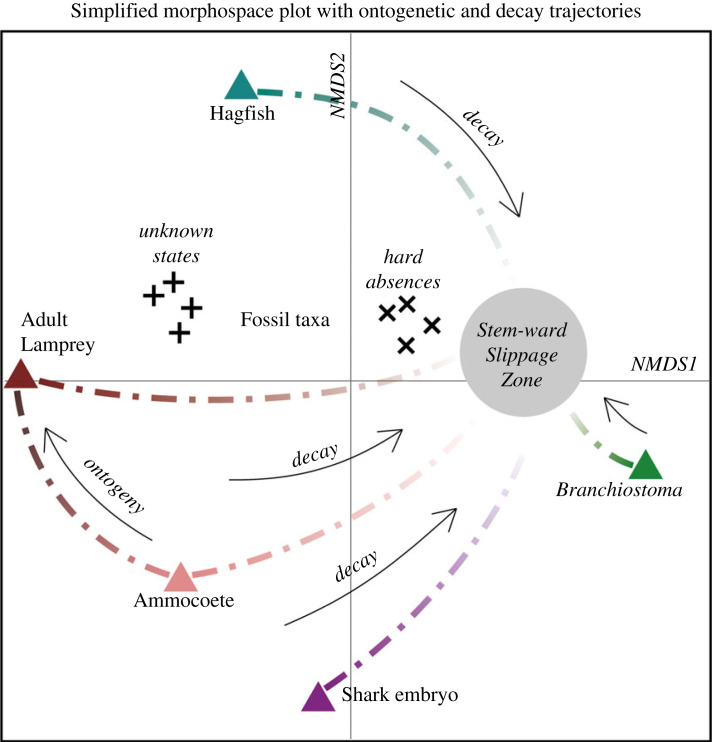
Illustrated summary of the key trends seen within the analysis. The two NMDS axes show clear separation of the semaphoronts and semataphonts. The complete modern taxa found are the extremities of the space. As organisms become more anatomically incomplete in successive day stages, they converge towards the centre of the space: the ‘stem-ward slippage zone’. The morphological transformation of lamprey during ontogeny (ammocoete to adult) runs in a direction perpendicular to the decay sequence. Black + and *x* symbols indicate the different coding styles applied to fossil taxa.

Here, we demonstrate a new multivariate approach to aid interpretation of problematic soft-tissue fossils by combining morphological data from extant taxa, decay data and fossil taxa to understand how different morphological influences may have impacted morphology preserved in fossils. Data from the extant organisms created a framework for comparison within the morphospace, highlighting areas of space that corresponded with morphological trait combinations under different conditions, in particular trait acquisition and transformation during ontogeny, and trait loss during decay (figure 3).

As anatomical traits are increasingly lost to decay, semataphonts begin to group closer together, converging in one area of the morphospace (figure 3). This produces a clear visualization of the stem-ward slippage phenomena [12]. Loss of more liable synapomorphic traits results in both a loss in variation and an increase in similarity between taxa (figure 2*a*). The convergence area can be inferred to represent an area of highly decayed morphology, but also an area that represents a more stem-ward plesiomorphic morphology. This secondary interpretation is supported by the occupation of *Branchiostoma*, a common proxy for an ancestral form (e.g. [40,78]), close to and within this region. For stem-ward slippage to operate in this way, whereby progressive trait loss through decay causes organisms to appear in a more ‘basal’ position phylogenetically speaking, there is a requirement for an approximate correlation between the relative resistance to decay of an anatomical trait and its propensity to become fossilized [11,31,32]. Of course there are preservational circumstances where this is not necessarily the case; for example, more decay-prone traits may in fact have higher preservation potential in circumstances where microbial-mediated authigenic mineralization is dominant [7,31,32,41,60,79]. Under such circumstances, the morphospace visualization technique applied here would still have utility for disentangling factors for anatomical variation, but a different approach to coding of the comparator semaphorants and semataphonts would be necessary. The data points would need to reflect the morphological variation under different conditions, e.g. retaining rather than removing the most decay-prone characters for analysis to reflect those specific taphonomic circumstances.

Ontogenetic changes have a less dramatic effect than decay-driven changes in our analysis. Ontogenetic factors resulted in only minimal separation of occupied space between the shark semaphoronts, but resulted in a clear, and expected, continuum of change within the lamprey from ammocoete larvae to the adult form (figure 2*a*). The ‘direction’ is consistent between taxonomic groups, related to changes in NMDS2. However, identification of this continuum of lamprey ontogenetic change is only possible due to our knowledge of their dramatic metamorphosis. Without the metamorphic stages to link the ammocoetes and adults, it is likely that their separation would be interpreted as separate taxonomic groupings within this analysis, as they were historically prior to the discovery of living metamorphic stages [80].

### (b) Evaluating affinities of fossil taxa through multivariate ordination

As expected, different fossil taxa exhibit morphological variation that is consistent with all three influential factors: ontogeny, taphonomy (in this case focused on decay-related incompleteness) and phylogeny. With respect to phylogeny, fossil taxa are morphologically close to a wide variety of extant forms. With respect to taphonomy, patterns of incompleteness in fossils are consistent with anatomical loss observed during decay. Finally, with respect to ontogeny, fossils group near to extant specimens that share similar interpreted ontogenetic development stages.

Of the three axes, the influence of ontogeny is the most subtle, but it is still clearly apparent within the data. *Priscomyzon* fossil specimens previously interpreted as subadult ontogenetic stages occupy areas close to the extant adult lamprey decay stages. This is consistent with the findings of Miyashita *et al.* [76], who describe the specimens of *Priscomyzon* as direct developers lacking metamorphic change, and as such, little difference would be expected between adult and subadult development stages. *Priscomyzon* also provides some indication of the interaction between factors. While some fossil specimens do group near the extant adult lamprey, several are positioned towards the extant semataphonts, indicating there may also be some influence of decay on these specimens. The specimen of *Mesomyzon,* a Cretaceous lamprey that was previously interpreted as a late metamorphic stage lamprey [81,82], is instead recovered here as being morphologically similar to early decay stage adult lampreys, while the ‘adult’ fossil specimen (labelled Mesomyzon_CJ) could also be considered as close to the extant late metamorphic stages. This was not expected, and potentially indicates some overlap in the tissues lost during decay and those developed during the final stages of metamorphosis, suggesting that taphonomic factors could have been conflated with ontogeny-derived change. However, it may also be indicative of the impact of evolution, creating morphological differences between extant lamprey and the *Mesomyzon* [83] that are hard to distinguish from taxonomically or ontogenetically linked changes. Without a model of decay of metamorphic lampreys, or more examples of early crown group lampreys, it is difficult to interpret further.

The impact of decay on the morphology of fossil taxa is striking throughout this analysis. The spectrum of incompleteness observed in fossil specimens of *Mayomyzon* parallels that seen with the increasing influence of decay on extant adult lampreys (figure 2*b*). The variability observed in specimens of *Mayomyzon* has previously been interpreted as related to incompleteness due to taphonomic factors, albeit under the influence of both decay and preservation factors [31]*.* The numbering system for the Gabbott specimens used in our analysis correlates to the ranked incompleteness described in Gabbott *et al*. [31], and we recover a similar order (with only minor deviations) to their position in the morphospace, consistent with a decay-related series (figure 2*b*, labelled as M.G1–20). A similar pattern is recovered with the series of Gabbott *Euphanerops* specimens (where variation is interpreted to have similar taphonomic drivers as their *Mayomyzon* specimens) with variation in morphology seeming to create a pattern within the plot that would be expected from a decay series (figure 2*d*, labelled as E.G1–17).

The phylogenetic structure of the underlying data is illustrated by the grouping of fossil taxa near to their extant forms, i.e. fossil lampreys specimens are positioned unambiguously near to extant lampreys, and *Tethymyxine*, which has clear diagnostic hagfish characteristics [66], is close to the extant hagfish regardless of coding style.

The positions of the other fossil specimens, *Palaeospondylus* and the ‘euphaneropoids’, are indicative of the difficulties of selecting comparative models, and the utility of this approach for supporting interpretations of affinity. When ambiguous coding states are permitted, *Palaeospondylus* anatomical ‘versions’ are generally recovered in morphospace positions close to the model taxon used to interpret their anatomy and homology (figure 2*e*). However, a stricter approach to coding characters results in a much tighter co-location of the various *Palaeospondylus* ‘versions’, in the area of relatively advanced decay and incompleteness (electronic supplementary material, figure S6). The dramatic sensitivity of *Palaeospondylus* to different coding approaches demonstrates how comparator groups can influence the assignment of affinity of a taxon, obscuring other influences on morphological variance. Given the different implications from these two approaches to character coding, it may be more beneficial to undertake future analyses using topological-only characters, an approach encouraged for assessment of problematic taxa [10,11,45]. In any case, the approach applied here serves as a powerful visualization of these different interpretation models simultaneously in terms of coherence and influence of phylogenetic, taphonomic and ontogenetic variability. The euphaneropoids (considered here as *Lasanius*, *Euphanerops, Jamoytius, Achanarella, Ciderius and Cornovichthys*) have been subject to various interpretations as stem-gnathostomes, stem-cyclostomes, anaspids or other, and as more or less closely related to each other (e.g. [11,43,66,77,84,85]). In both coding approaches, they are morphologically close to the decay stages of extant adult lampreys (figure 2*d*; electronic supplementary material, figures S1, S4, S6 and S9). This closeness between fossil ‘euphaneropoids’ and lampreys, could be interpreted as affinity between the groups, providing support to several previous analyses of this group [45,66,77], although it is by no means a well-agreed-upon position [11,27,85–87]. As demonstrated with *Palaeospondylus*, model selection can have an impact on the interpretation of affinity. Where a lamprey model is used for morphological interpretation, the resulting *Euphanerops* variant can be found to be morphologically close to the lamprey semataphonts (labelled Euphanerops_2018); using an alternative anatomical interpretation (using the Anaspida as a comparative model [88]) *Euphanerops* is found in a more ambiguous cyclostome position between the hagfish and lampreys. This does not rule out lamprey affinity for *Euphanerops*, but simply illustrates the impact that model selection can have on morphological interpretation.

### (c) Applicability of multivariate analyses to fossil interpretations

Here, we have demonstrated how this multivariate approach enables testing of fossil affinity by simultaneously visualizing multiple axes of morphological variation (i.e. taphonomic, ontogenetic and phylogenetic). This method is intended as an exploratory approach to reduce the uncertainty of the impact of multiple sources of variation, which would allow for more confident anatomical interpretations. Although here we have used early vertebrate fossils to highlight the potential application and limitations of this method, ordination of semaphoronts and semataphonts is not just applicable to this group. The same or a similar method will be applicable to other similar examples of enigmatic or previously intractable soft-bodied fossil taxa, including analysis of fossil specimens of the same taxon subjected to different preservation modes, or even to biomineralizing groups where ontogenetic and/or taphonomic factors have obscured interpretations. Future analyses of this kind may need to consider the following:

First, as with other morphological comparisons, the initial character or trait list should be carefully considered to adequately capture the similarities/dissimilarities between taxa. In all analyses, careful consideration is needed with respect to the impact of initial data sampling. This is especially important with respect to ordination studies such as those applied here, because the choice and scope of taxa and traits selected for inclusion will have a big effect on the outcomes, given the potential amplification or dissipation of the similarities and dissimilarities, and the distribution of data points in the ordination space.

In this study, the character list was informed in large part by trait lists used in taxonomy and comparative taphonomy [12,41,42], and as a result, lacked information on traits that inform on other aspects (i.e. ontogenetic change). This may have led to omission of some informative and diagnostic ontogenetic traits (e.g. changing fin number, size and shape), and decay-resistant features (such as biomineralized structures other than teeth). However, care also needs to be taken not to over-represent traits only regularly seen in extant taxa (e.g. colour changes) which may force false disparity into the dis/similarity matrix. Ideally the character list should be bespoke for the organisms included in it, which would also allow for the design of taphonomic experiments to capture a greater amount of detail. Broadening the range of traits included will also have the effect of potentially artificially amplifying similarities between data points if, for example, those characters are invariant (or not observed) for a range of semaphoronts or semataphonts (unlike in phylogenetic analyses).

Secondly, the different approaches to coding were found to have a significant impact on interpretations of fossil taxa. ‘Unknown’ entries (i.e. ‘?’ character state) represent a pre-assumed bias within the dataset, and should be avoided. However, a strict approach creates an inflexible method that fails to adequately account for taxa that are only known from clearly damaged or incomplete fossils. In such cases, it may be prudent to include a more flexible approach which would explicitly encode such unknown or ambiguous character entries when comparative taphonomy indicates that a feature might be present but not preserved. However, this approach risks circularity given that the approach outlined here is to serve as an independent approach to comparative taphonomy.

Third, the inclusion of more non-ambiguous fossil data may help improve the robustness of the comparative framework. A notable absence in this work is the stem-gnathostomes, represented in the fossil record by a large selection of well-preserved specimens. However, caution is also needed in this approach as inclusion may be biased by preconceptions of affinity, so a range of different fossil groups is recommended.

Finally, the taphonomic aspect of this study is focused on decay-induced incompleteness; however, many other processes are involved, which need to be considered to create robust interpretations. For example, preservational mechanisms, in particular how they operate and vary between sites, and how they interact with decay processes, are a crucial and as yet missing part of the puzzle. Here, they are only captured partially within the Gabbott series of *Euphanerops* and *Mazomyzon* [31] (i.e. combinations of organic, phosphatization and pyritization pathways)*.* In our analysis, the decay-related semataphonts are theoretical constructs based on a relatively straightforward paradigm whereby we expect a linear loss of traits as organisms are subjected to more decay over time [12,31]*.* An alternative approach to consider different preservation pathways under different conditions could be applied, but would be difficult to achieve without a better understanding of how morphology is expected to be preserved in the same taxon under different taphonomic conditions. Explicit inclusion of fossils preserved in different preservation pathways (e.g. a pyritized semataphont and a phosphatized semataphont) would be a powerful way to broaden the visualization of taphonomic variability in the ordination space. However, to fully integrate more taphonomic factors, a clearer understanding about the relationship between decay sequences, tissue composition, and tissue preservation in laboratory and real-world empirical settings would help [30]*.* Where this data is available, inclusion into the analysis would be relatively simple to achieve. The extant semaphorants represent the ‘complete’ morphology, providing a reference from which additional theoretical semataphonts could be created by ‘editing’ the semaphorant morphology. The rules for how traits would be ‘edited’ need codifying *a priori* (e.g. iron-rich anatomical features that decay quickly, like nervous tissues, could have enhanced preservation in some circumstances [89]). This could also be expanded to include [63] the preservational biases found between sites (e.g. [7]) where groups of different semaphorants and semataphonts would be created for each site. Alongside preservational mechanisms, other taphonomic factors need to be considered; sources such as transportational damage [63], and post-fossilization processes, like weathering [90], can have considerable impact on fossil fidelity.

The work presented here is intended as a demonstration of the overall approach to using extant data to explore the underlying drivers of morphological variation in fossils. The use of a well-researched case study group allowed us to test the utility of the method, and explore its potential use in other problematica, including mineralized taxa. Our taphonomic axis is focused on the impact of decay but conclusions without a greater taphonomic context must be treated with caution. However, the data in this area are limited. To strengthen any future application of this method, or indeed any further analyses into soft-tissue fossils, further experimental taphonomy and preservational investigations are needed. This includes understanding how decay develops under different environmental conditions, and how different modes of preservation might interact with different tissues and over different time-frames and rates. It is also evident that more work is needed in understanding how well data from *in vivo* experiments reflect real fossilization conditions [31,32,60,65].

## 6. Conclusion

Visualizing morphological data makes understanding the trait combinations and recognizing the potential biases within the fossils easier, creating a guide to interpretations. In this study, we present a new exploratory approach to using extant data to inform on interpreting problematic taxa. In our case study with enigmatic early vertebrate fossils, we find all three axes of morphological variation (i.e. ontogenetic, taphonomic and phylogenetic) to be important and interdependent factors; we interpret the variability of the fossil lamprey *Mayomyzon* to be consistent with spectra of incompleteness resulting from variable degrees of decay [31], the variability of *Priscomyzon* to be consistent with a direct-developing lamprey rather than pre-metamorphosis lamprey [76], and the presumed phylogenetic variability between different ‘euphaneropoid’ taxa to be limited with the taxa clustering together, close to a cyclostome affinity*.* That multivariate analyses find the influence of all sources of morphological variation to be simultaneously present underscores the power of this kind of approach for analysis of problematic or enigmatic fossil taxa: if only one axis is considered at a time (e.g. taphonomy or ontogeny), then the important role of another axis will be neglected and potentially fail to be recognized or accounted for. Furthermore the important role that choice of extant comparator organism plays is demonstrated given the highly variable placement of the problematic taxon *Palaeospondylus*. The approach outlined here enables visualization of different approaches to interpreting and homologizing fossil anatomy, evaluating them in terms of their coherence, and the role of ontogenetic and taphonomic variability. Although non-biomineralized vertebrates were used as a case study, we feel that by careful selection of morphological influences, and by building a comparative framework appropriately, this phenetic exploratory method has potential applications with other problematica throughout the fossil record, not just soft-tissue taxa.

## Data Availability

Data are available from the Dryad Digital Repository: https://doi.org/10.5061/dryad.stqjq2c7b [91]. The data are provided in electronic supplementary material [67].
